# Metformin Inhibits ROS Production by Human M2 Macrophages via the Activation of AMPK

**DOI:** 10.3390/biomedicines10020319

**Published:** 2022-01-29

**Authors:** Rana M. Nassif, Elias Chalhoub, Pia Chedid, Margarita Hurtado-Nedelec, Elia Raya, Pham My-Chan Dang, Jean-Claude Marie, Jamel El-Benna

**Affiliations:** 1 Faculty of Health Sciences, University of Balamand, P.O. Box 55251 Sin El Fil, Beirut 1100-2807, Lebanon; rana.nassif@balamand.edu.lb (R.M.N.); elias.chalhoub2@gmail.com (E.C.); piachedid@gmail.com (P.C.); elia.raya@gmail.com (E.R.); 2 Centre de Recherche sur l’Inflammation (CRI), Laboratoire d’Excellence Inflamex, Faculté de Médecine Xavier Bichat, Université de Paris, INSERM-U1149, CNRS-ERL8252, 75018 Paris, France; maria.hurtado-nedelec@aphp.fr (M.H.-N.); my-chan.dang@inserm.fr (P.M.-C.D.); jean-claude.marie@inserm.fr (J.-C.M.)

**Keywords:** metformin, macrophage, inflammation, ROS, NADPH oxidase, NOX2, AMPK

## Abstract

Metformin (1,1-dimethylbiguanide hydrochloride) is the most commonly used drug to treat type II diabetic patients. It is believed that this drug has several other beneficial effects, such as anti-inflammatory and anticancer effects. Here, we wanted to evaluate the effect of metformin on the production of reactive oxygen species (ROS) by human macrophages. Macrophages are generated in vivo from circulating monocytes depending on the local tissue environment. In vitro proinflammatory macrophages (M1) and anti-inflammatory macrophages (M2) can be generated by culturing monocytes in the presence of different cytokines, such as GM-CSF or M-CSF, respectively. We show that metformin selectively inhibited human monocyte differentiation into proinflammatory macrophages (M1) without inhibiting their differentiation into anti-inflammatory macrophages (M2). Moreover, we demonstrate that, in response to LPS, M2 macrophages produced ROS, which could be very harmful for nearby tissues, and metformin inhibited this process. Interestingly, metformin with LPS induced activation of the adenosine-monophosphate-activated protein kinase (AMPK) and pharmacological activation of AMPK by AICAR, a known AMPK activator, decreased ROS production, whereas the deletion of AMPK in mice dramatically enhanced ROS production in different types of immune cells. These results suggest that metformin exhibits anti-inflammatory effects by inhibiting the differentiation of human monocytes into M1 macrophages and by limiting ROS production by macrophages via the activation of AMPK.

## 1. Introduction

Metformin, also known as 1,1-dimethylbiguanide hydrochloride, belongs to the family of biguanides [1,2]. It is the first drug of choice for the treatment of type II diabetic patients [3,4]. Metformin acts as an insulin sensitizer and decreases gluconeogenesis, especially in the liver, while it increases glucose uptake by skeletal muscle cells. Metformin also improves the use of glucose by fat cells. The result of metformin’s uptake is the decrease in circulated glucose in the blood [5]. At a cellular level, studies showed that metformin increased AMPK activity in hepatocytes [6]. AMPK is a phylogenetically conserved serine/threonine protein kinase [7,8]. The kinase is a complex heterotrimeric protein composed of three subunits: a catalytic α subunit and two regulatory β and γ subunits [1,9]. In fact, it is currently known that metformin enters the cell using organic cation transport (OCT) [10]. Then, it acts first by inhibiting the complex I of the electron transport chain located at the internal membrane of the mitochondria, leading to the decrease in ATP production [11,12], and second by activating the tumor suppressor liver kinase I B1 (LKB1), which phosphorylates AMPK [13]. The transient reduction in cellular energy (a decrease in ATP levels versus an increase in AMP levels), as well as the phosphorylation of AMPK both activate switching of the cell’s status from anabolic to catabolic [1,14,15]. AMPK regulates the metabolism and probably other cellular functions by linking them with the available energy levels in the cell [16]. Indeed, it is implicated in inflammation, as increased inflammatory markers in both mouse models of obesity [17] and in obese humans [18] are associated with an attenuation of AMPK phosphorylation.

Increasing evidence suggests that metformin exhibits anti-inflammatory, antioxidant, and anticancer effects, which are beyond its established pharmacological and cellular actions [2,3,4]. Indeed, it was shown that KRAS mutants promote metabolic dysregulation in cancer cells [19] and that metformin selectively inhibits metastatic colorectal cancer with the KRAS mutation [20]. Interestingly, pharmacological activation of AMPK by 5-aminoimidazole-4-carboxamide-1-β-4-ribofuranoside (AICAR) enhances the efficacy of rapamycin, the mTORC1 inhibitor, to kill human cancer cells by regulating phospholipase D (PLD) activity [21]. These data suggest that pharmacological activation of AMPK by metformin could be an interesting strategy in cancer treatment and the treatment of other diseases.

Several key regulatory inflammatory roles are attributed to macrophages, which are heterogeneous cells present in lymphoid and nonlymphoid tissues [22]. They are very efficient phagocytic cells because they have multiple receptors capable of recognizing a large set of pathogens [23]. The colony-stimulating factor (CSF) plays an essential role in the process of differentiation of circulating monocytes into macrophages [24], and their exposition to a combination of cytokines induces their polarization into different macrophages exhibiting different patterns [22]. In fact, monocytes can differentiate into classically activated macrophages, also known as M1 macrophages. These macrophages have proinflammatory functions, such as phagocytosis accompanied by reactive oxygen species (ROS) production [25], cytolytic and proteolytic activities, removal of damaged tissues known as M2 macrophages. These macrophages exhibit anti-inflammatory functions, such as tissue repair and remodeling, angiogenesis, and immunosuppression [26], and show different transcriptional profiles depending on their localization [27]. They are either involved in the immune response against pathogens (generation and resolution of inflammation) or the surveillance of tissue changes and body homeostasis through phagocytosis of apoptotic cells and the production of growth factors [23]. Thus, a regulated ratio of M1 versus M2 macrophages is important for appropriate inflammatory responses.

Likewise, a regulated production and release of ROS by phagocytes is needed to cope with host infection and subsequent resolution. ROS are small intensely active molecules deriving from the reduction in molecular oxygen and include superoxide anion (O_2_^−^), hydroxyl radical (OH°), hydrogen peroxide (H2O2), and hypochlorous acid (HOCl) [28]. In phagocytes, ROS are generated by the nicotinamide adenine dinucleotide phosphate (NADPH) oxidase (NOX2) and the myeloperoxidases (MPO) [29]. During the process of phagocytosis, recognized foreign molecules are internalized into phagocytic vacuoles and are in contact with the intracellular ROS [30]. During this phenomenon, ROS generated by an oxidative burst are essential for the degradation of the internalized particles [31]. Into phagosomes, the NADPH oxidase NOX2 plays an essential role in producing ROS. This is sustained by patients suffering from chronic granulomatous disease (CGD), a genetic disease characterized by a lack of NOX2 activity. These patients suffer from recurrent bacterial and fungal infections, leading to a continuous inflammatory status [28,32]. NOX represents a family of seven transmembrane enzymes: NOX1, NOX2, NOX3, NOX4, NOX5, DUOX1, and DUOX2 [33]. The expression of these proteins differs according to the cellular type and the environmental conditions [30]. Among the NOX family, NOX2 is predominantly expressed in immune cells, particularly in neutrophils, monocytes, and macrophages [34]. It is a multi-protein complex composed of two membrane subunits that constitute the catalytic unit of the enzyme (gp91^phox^ and p22^phox^) and four cytoplasmic subunits constituting the regulatory component of the complex (p67^phox^, p47^phox^, p40^phox^, and Rac1) [35]. The associated ROS production can be modulated in the presence of bacterial agonists, such as fMLF, LPS, and cytokines, such as TNF-α and IL-1β [36,37]. Several protein kinases, such as MAP kinases (ERK1/2 and p38) and PKC, can phosphorylate the p47^phox^ regulatory sub-unit on its serine residues located in its C-terminal end. This results in the migration of all the cytosolic sub-units to the plasma membrane [38,39,40]. In fact, the activation of the NOX2 complex relies on the association of all the subunits at the plasma membrane [41].

It is well established that ROS are involved in biological and physiological functions other than microbial killing. Indeed, an imbalance between ROS production and removal may induce oxidative stress and tissue damage [42,43]. This can disturb cellular and tissue homeostasis, resulting in pathologic status, such as inflammation, atherosclerosis, and cancers [28,30,44]. Here, we hypothesized that metformin exerts its anti-inflammatory and antioxidant effect by modulating macrophage polarization and by affecting ROS production in an AMPK-dependent manner. Consequently, we first examined the effect of metformin on human macrophage polarization. Then, we studied the effect of metformin on ROS production and the potentially involved signaling pathway in M2 macrophages. We found that metformin decreases M1 macrophage polarization, while maintaining M2 macrophage polarization, and reduces ROS production by these macrophages. These results suggest that metformin may exhibit anti-inflammatory and antioxidant effects by respectively inhibiting the differentiation of human M1 versus M2 macrophages and ROS production via the activation of AMPK.

## 2. Material and Methods

### 2.1. Chemicals and Reagents

Metformin was supplied from Benta Pharma Industries, DbayehLebanon. Lipopolysaccharide (LPS), *N* formyl-methionyl-leucyl-phenylalanine (fMLF), 5-aminoimidazole-4-carboxamide ribonucleotide (AICAR), luminol (5-amine-2,3-dihydro-1,4-phtalazinedione), horseradish peroxidase (HRPO), nitro blue tetrazolium chloride (NBT), Hanks’ balanced salt solution (HBSS), and Dulbecco’s phosphate-buffered saline (DPBS) were purchased from Sigma Aldrich (Saint-Quentin Fallavier, France). Dimethyl sulfoxide (DMSO) was purchased from Honeywell Research chemicals. Dextran T500 and Ficoll were purchased from GE Healthcare (Orsay, France). RPMI 1640 medium, fetal bovine serum (FBS), and penicillin/streptomycin solution were purchased from Invitrogen (Life Technologies, Asnières-sur-Seine, France). Granulocyte macrophage colony-stimulating factor (GM-CSF), macrophage colony-stimulating factor (M-CSF), and interleukin-4 (IL-4) were purchased from Peprotech (Neuilly-Sur-Seine, France).

### 2.2. Ethics Statement and Monocytes Isolation

Monocytes were isolated from healthy volunteers’ venous blood after their informed consent. The collection and analyses of data were performed anonymously. The study was approved by the institutional review board of Inserm and ethics committee of the Lebanese hospitals. Blood units were collected from different hospitals located in Beirut, Lebanon and Paris, France. Human peripheral blood mononuclear cells (PBMC) were isolated using the Ficoll/dextran technique. In brief, 2% dextran was mixed with a blood volume and kept at 4 °C, allowing red blood cell sedimentation. The supernatant was then layered on Ficoll (ratio 2:1). Following centrifugation, the PBMC ring was collected and washed twice in phosphate buffer saline, then the cell pellet was resuspended in PBS + BSA 0.5% + 1 mM EDTA. CD14+ cells were isolated following the kit manufacturer’s protocol (Easy Sep human monocyte enrichment kit from Stem Cell). In brief, PBMC were transferred in a 14 mL polystyrene round-bottom tube (BD Biosciences, Rungis, France) at a density of 5 × 10^7^ cells/mL and incubated first with a cocktail of antibodies, followed by a second incubation with magnetic beads. The tube containing the mixture was then held open in the stem cell magnet for approximately 3 min at room temperature and monocytes were recovered by negative selection in a 15 mL tube and washed with PBS.

### 2.3. Human Monocyte Differentiation and Polarization

For the differentiation and polarization of isolated human monocytes into M1 and M2 macrophages, cells were cultured for 7 days at 37 °C in a 5% CO_2_ atmosphere in complete medium composed of RPMI 1640 medium supplemented with 10% fetal bovine serum and 1% penicillin–streptomycin solution in the presence or absence of different cytokines. Monocytes were seeded in 96- or 6-well culture plates at 2 × 10^5^ cells/well or 1 × 10^6^ cells/well, respectively, and the following cytokines were added for 7 days: 50 ng/mL GM-CSF (for M1 polarization) or 50 ng/mL M-CSF (for M2 polarization). At day 6, 100 ng/mL LPS was added to M1 polarized macrophages and 20 ng/mL IL-4 was added to M2 polarized macrophages. Seven days later, cells were detached from plates and stained with a cocktail of conjugated antibodies as follows: 10 µL CD14-FITC, 5 µL CD16-APC-H7, 5 µL CD80-PE-Cy5, 5 µL CD163-APC, and 5 µL CD200R-PE from BD Biosciences. Data acquisition was performed on 10,000 cells with constant PMT values on an FACS Canto II cytometer (BD Biosciences). Data analysis was carried out using Diva software (BD FACSDiva v9.0software, BD).

### 2.4. Monocyte and Macrophage Cytotoxicity Tests

Monocytes or macrophages were seeded in 96-well plates at 10^5^ cells/well in complete medium. Metformin treatment was added at different concentrations (0.5, 1, and 2 mM) to monocytes, and the plates were incubated for 24, 48, and 144 h at 37 °C in 5% CO_2_. For M2 macrophages, 4 μg/mL LPS and 2mM metformin treatments were added, and the plates were incubated for 3 h at 37 °C in 5% CO_2_. Then, cytoX reagent was added to the cells and incubated for 30 min. The absorbance was measured at 450 nm using a microplate reader from Thermo Scientific, Asnières-sur-Seine, France).

### 2.5. Reactive Oxygen Species (ROS) Measurement by the NBT Reduction Assay

Reactive oxygen species (ROS) were measured using nitro blue tetrazolium chloride (NBT) reduction assay. Human isolated monocytes were seeded in 96-well plates at 2 × 10^5^ cells/well. After 7 days, 4 μg/mL LPS and metformin were applied to the M2 macrophages and the plates were incubated for 1 and 3 h at 37 °C in 5% CO_2_. Then, 55 min after incubation, 0.1 mg/mL NBT was added in the dark, followed by the addition of the solubilization solution (80% 1M KOH and 20% dimethyl sulfoxide) to dissolve formazan crystals. Absorbance was then measured at 570 nm using a microplate reader from Thermo Scientific).

### 2.6. Luminol-Amplified Chemiluminescence Assay

A total of 5 × 10^5^ cells/mL were suspended in Hank’s balanced salt solution (HBSS) with 10 µM luminol and 2.5 U horseradish peroxidase. Similar treatments (4 μg/mL LPS +/− ± 2 mM metformin) were applied on cells, followed by the addition of 10^−6^ M fMLF. Luminol-amplified chemiluminescence was then measured using the luminometer (AutoLumat lb 953, Berthold Technologies Thoiry, France) for 15 min and kinetic curves were generated in counted photons per minute (cpm).

### 2.7. Animal Experiments

Animal studies were performed in accordance with the European Community Guidelines. All protocols were approved by the Ethics Committee for Animal Research of University of Paris and INSERM (CEEA-JCM.121). AMPKα1–/– mice were a generous gift of Dr. Benoit Viollet (Institut Cochin, Paris, France) [45]. Blood was withdrawn from wild-type and AMPKα1–/– mice tails using heparin as the anticoagulant. For the isolation of bone marrow cells, femurs and tibiae of 12- to 16-week-old mice were flushed with sterile HBSS containing 1 mM EDTA+ 0.1% albumin; cells were counted and used for luminol-amplified chemiluminescence as described above.

### 2.8. Western Blot Analysis

Human isolated monocytes were seeded in 6-well plates at 1 × 10^6^ cells/well. After 7 days, 2 mM metformin and 4 µg /mL LPS were applied to the cells for 1 and 3 h in Hank’s balanced salt solution (HBSS). The incubation was carried out at 37 °C in 5% CO_2_. Proteins were extracted from cells using 1X Laemmli sample buffer containing 50% glycerol, 12.5% sodium dodecyl sulfate (SDS), 25% beta-mercaptoethanol, 312.4 mM Tris-HCl pH 6.8, 12.5 mM EDTA, 12.5 mM EGTA, 0.75 mM bromophenol blue, protease, and phosphatase inhibitors. Lysates were then incubated at 95 °C for 15 min and stored at −80 °C. Proteins were separated on 10% SDS-polyacrylamide gels using a Bio-Rad system, then transferred on nitrocellulose membrane (Amersham, UK, GE Healthcare). Membranes were blocked in TBS-Tween (20 mM Tris-HCl, pH 7.6, 137 mM NaCl, 0.1% Tween 20) containing 5% non-fat dry milk for 1 h at room temperature. They were then incubated overnight at 4 °C with the following primary antibodies: mouse monoclonal p22^phox^ (1:2000 from Santa Cruz, Heidelberg, Germany), rabbit monoclonal p47^phox^ and p67^phox^ (1:4000, manufactured in-house [39,46]), mouse monoclonal gp91^phox^ (1:4000 from Santa Cruz), rabbit monoclonal AMPK and phospho-AMPK (1:1000 from Cell Signaling, Ozyme, Saint-Cyr-l'École, France), rabbit monoclonal phospho-p38MAPK (T180/Y182) (1:1000) (Cell Signaling), rabbit monoclonal phospho-ERK1/2 (T202/Y204) (1:1000) (R&D Bio-Techne, Minneapolis, USA), mouse monoclonal GAPDH and β-actin (1:2000 and 1:1000 from Santa Cruz), phospho-Ser345P-p47^phox^ and phospho-Ser328P-p47^phox^ (1:10,000 and 1:2500, manufactured in-house [39]). After washes with TBS-Tween, membranes were incubated with goat anti-mouse or anti-rabbit (1:10,000) horseradish peroxidase-conjugated secondary antibodies for 1 h at room temperature. After several washes, membranes were revealed using an ECL solution (GE Healthcare LifeSciences, Velizy, France) and visualized using Amersham Imager 600 (GE Healthcare LifeSciences).

### 2.9. Statistical Analysis

Data were expressed as means ± SD, which were analyzed with SPSS statistics software from triplicates. Multiple comparisons were determined by paired sample *T*-test using the SPSS software SPSS Statistics software version 28.0.1.0 (142). Results were considered significant when the *p* value was below 0.01 and 0.05.

## 3. Results

### 3.1. The Effects of Metformin on the Polarization of Human Monocytes into M1 and M2 Macrophages

**Figure 1:**
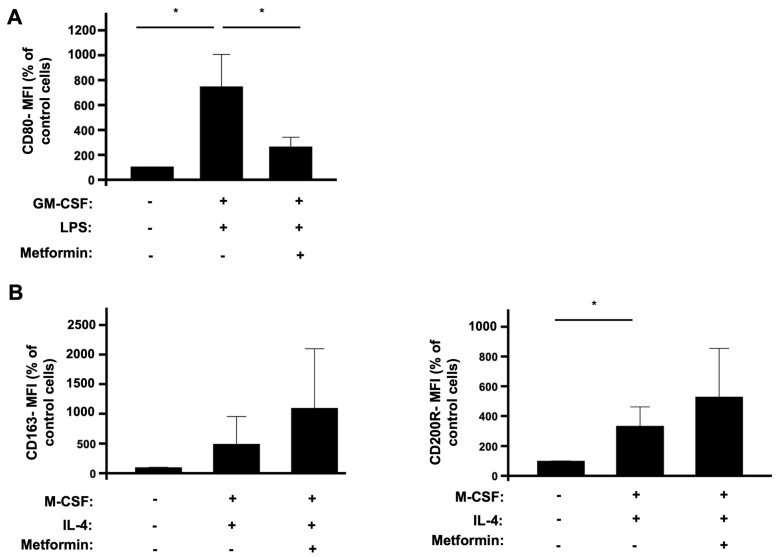
Effect of metformin on human isolated monocytes’ polarization into M1 and M2 macrophages. Human monocytes were isolated from blood of healthy donors and incubated in the absence or presence of metformin (2 mM) and GM-CSF (50 ng/mL) and LPS (100 ng/mL) for 7 days and analyzed by flow cytometry using fluorescent anti-CD14, anti-CD16, and anti-CD80 antibodies (**A**). Human monocytes were isolated from blood of healthy donors and incubated in the absence or presence of metformin (2 mM) and M-CSF (50 ng/mL) and IL-4 (20 ng/mL) for 7 days and analyzed by flow cytometry using fluorescent anti-CD14, anti-CD16, and anti-CD163 and anti-CD200R antibodies (**B**). Cell sorting and profiling was completed using a FACS Canto II cytometer and DIVA software for analysis. Data are mean of fluorescence (n = 3). * *p* value < 0.05.

To investigate the effect of metformin on ROS production by human macrophages, we first wanted to check its effect on the polarization of monocytes into M1 and M2 macrophages. Monocytes were isolated from healthy human blood, polarized into classically M1 macrophages using GM-CSF and LPS, and into nonclassically M2 macrophages using M-CSF and IL-4. The effect of metformin on the polarization of monocytes into macrophages was estimated by sorting and profiling macrophage markers using flow cytometry. Double-positive CD14 and CD16 cells were profiled as monocytes [26,47]. Among these, we categorized M1 polarized macrophages as CD14+, CD16+, and CD80+ cells, whereas M2 polarized macrophages were CD14+, CD16+, CD163+, and CD200R+. Cells which had no cytokines treatment were the control group. As expected, the combination of GM-CSF and LPS significantly induced the polarization of monocytes into M1 macrophages (Figure 1A), while the combination of M-CSF and IL-4 induced the polarization of monocytes into M2 macrophages (Figure 1B). Most importantly, when incubated with cytokines in the presence of metformin, monocyte polarization towards M1 macrophages was significantly decreased (Figure 1A), whereas metformin did not decrease the polarization of monocytes into M2 macrophages, but rather it has the tendency to increase it (Figure 1B).

### 3.2. Metformin Inhibits Reactive Oxygen Species (ROS) Production in M2 Macrophages

**Figure 2:**
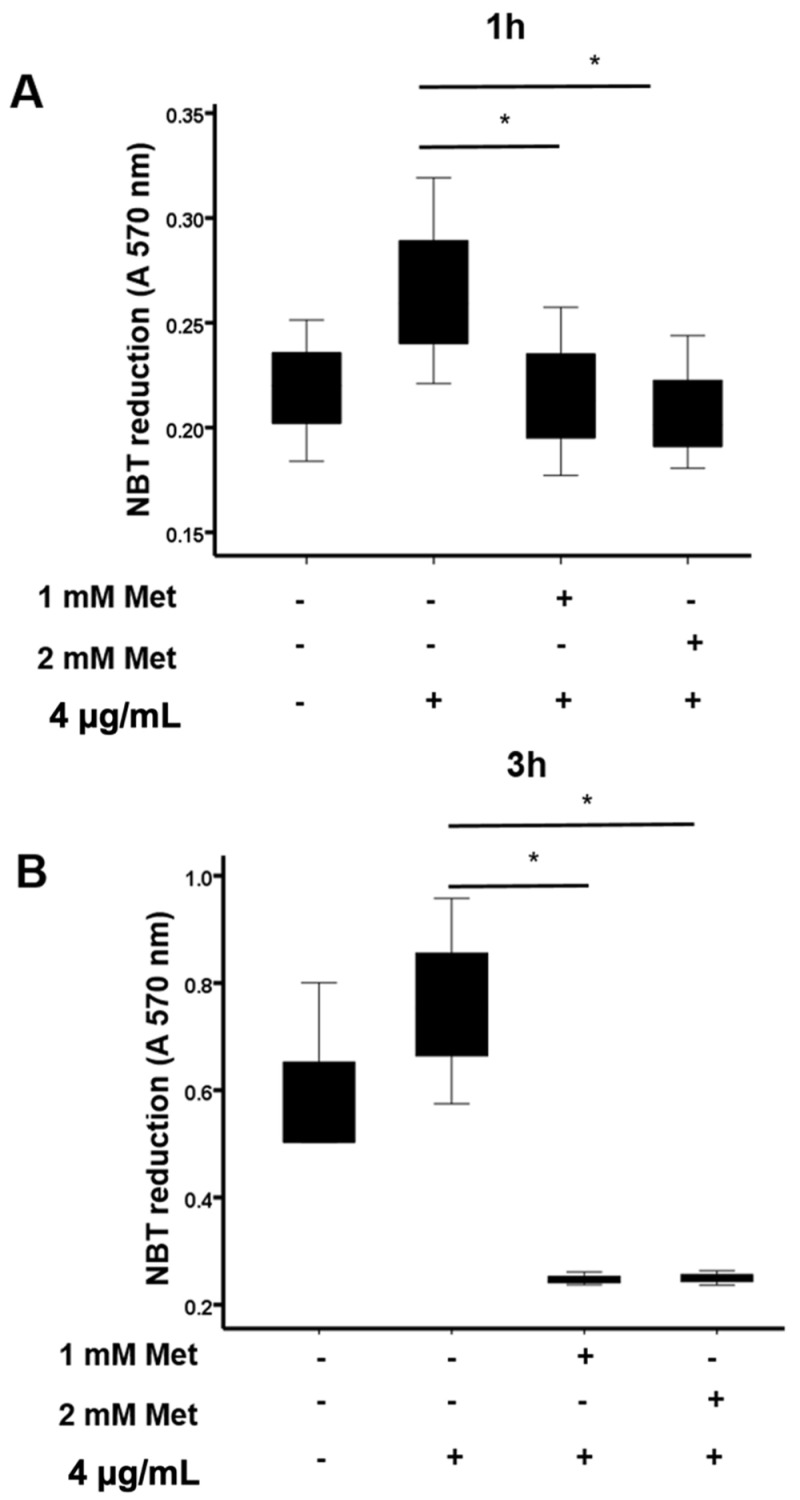
Effect of metformin on ROS production in M2 polarized macrophages. M2 macrophages were incubated in the absence or presence of metformin (1 and 2 mM), then stimulated by LPS (4 µg/mL) for 1 (**A**) and 3 h (**B**). The relative intracellular ROS production was determined by recording the reduction in NBT at 570 nm. Data are mean of O.D. ± SD (n = 3). * *p* value < 0.05.

The above results showed that metformin inhibited M1 macrophage generation, while favoring M2 macrophages generation. M2 macrophages can produce reactive oxygen species (ROS), which are able to induce oxidative stress and tissue injury. We thus tested the effect of metformin on ROS production by M2 macrophages. These macrophages were incubated with LPS in the absence or presence of 1 and 2 mM metformin for 1 and 3 h. Then, ROS production was measured by the NBT reduction assay at 570 nm. Macrophages without any LPS stimulation were considered as the control group. Results show that LPS stimulated ROS production by M2 macrophages and that metformin treatment significantly inhibited LPS-induced ROS production in these macrophages after 1 and 3 h (Figure 2A,B). Taken together, these data show that metformin did not affect monocyte polarization to M2 macrophages and prevented their ROS production.

### 3.3. Metformin Does Not Affect Cell Viability

**Figure 3:**
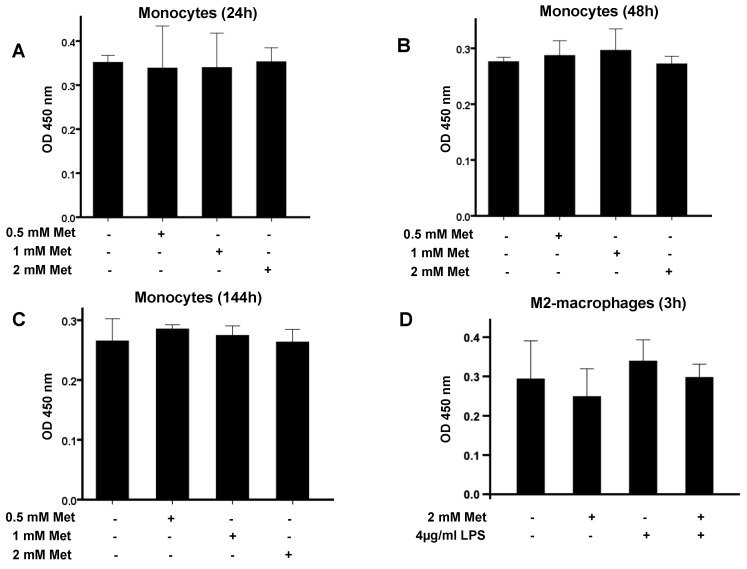
Evaluation of the cytotoxic effects of metformin on monocytes and M2 polarized macrophages. Human monocytes (**A**–**C**) and M2 macrophages (**D**) were incubated in the absence or the presence of metformin (0.5, 1, and 2 mM) for different indicated time periods. Cytotoxicity was analyzed after the addition of cytoX reagent and measurement of absorbance at 450 nm. Data are mean of absorbance ± SD (n = 3).

To verify that the observed inhibitory effects of metformin on monocytes’ differentiation into M1 macrophages and on ROS production by M2 macrophages were not due to a cytotoxic effect, monocytes and M2 polarized macrophages were incubated with metformin. We then measured the cytotoxic effects at 450 nm after the addition of cytoX reagent. Monocytes and M2 polarized macrophages without any treatment were considered as the control group. Results show that metformin did not affect monocyte and macrophage viability (Figure 3A–D).

### 3.4. Metformin Does Not Affect the Expression of the NADPH Oxidase/NOX2 Components nor the Phosphorylation of p47^phox^ on Ser345 and Its Upstream Kinases p38 and ERK1/2

**Figure 4:**
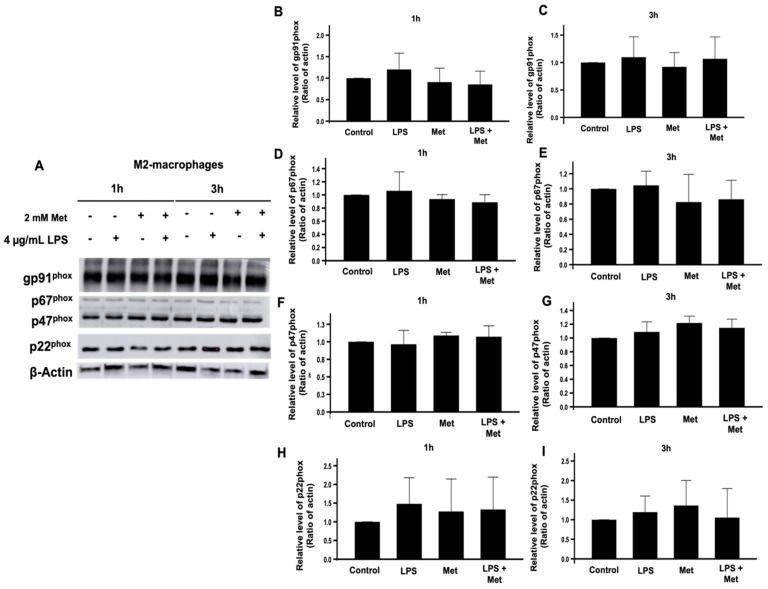
Effect of metformin on the expression of the NADPH oxidase components. M2 macrophages were incubated in the absence or the presence of 2 mM metformin then stimulated by LPS (4 µg/mL) for 1 and 3 h. The expression of gp91^phox^, p22^phox^, p47^phox^, and p67^phox^ was evaluated compared to actin by SDS-PAGE and Western blot using specific antibodies (**A**). The gp91^phox^ (**B**,**C**), p67^phox^ (**D**,**E**), p47^phox^ (**F**,**G**), p22^phox^ (**H**,**I**) and actin bands from three experiments were quantified and expressed as mean ± SD.

**Figure 5:**
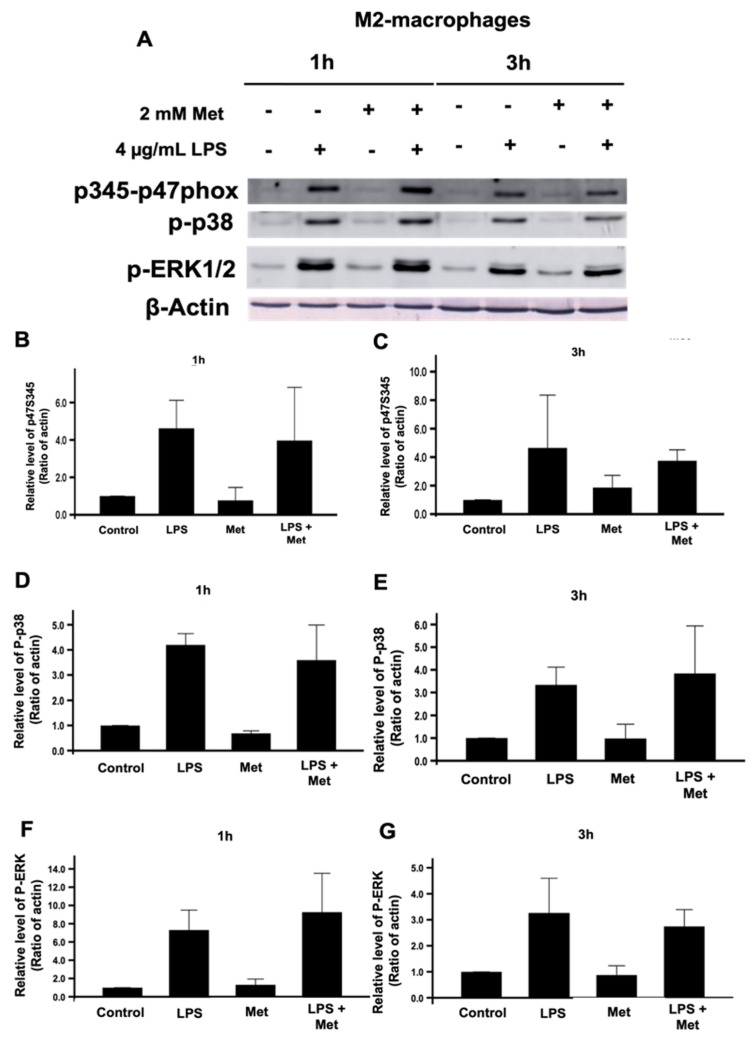
Effect of metformin on the phosphorylation of Ser345 of p47phox and its upstream MAP kinases, p38MAPK, and ERK1/2 in M2 macrophages. M2 macrophages were incubated in the absence or the presence of 2 mM metformin, then stimulated by LPS (4 µg/mL) for 1 and 3 h. The phosphorylation of Ser345-p47phox, p38MAPK, and ERK1/2 was evaluated compared to actin by SDS-PAGE and Western blot using specific antibodies (**A**). The pSer345-p47phox (**B**,**C**), p38MAPK (**D**,**E**), and ERK1/2 (**F**,**G**) and actin bands from three experiments were quantified and expressed as mean ± SD.

To investigate if the inhibitory effect of metformin on ROS production by M2 macrophages is due to the inhibition of the expression of the NOX2 subunits, gp91^phox^, p22^phox^, p47^phox^, and p67^phox^ or not, we studied the effect of metformin on the expression of these proteins by SDS-PAGE and Western blots. Results show that metformin did not affect gp91^phox^, p22^phox^, p47^phox^, and p67^phox^ levels in M2 macrophages (Figure 4). As the activation of NOX2 is dependent on the phosphorylation of the p47^phox^ on its Ser345 by MAP kinases (p38 and ERK1/2), we evaluated the effect of metformin on these pathways. Results show that metformin had no effect on the phosphorylation of p47^phox^ on Ser345, nor on the p38 and ERK1/2 phosphorylation levels (Figure 5).

### 3.5. Metformin and LPS Enhanced AMPK Phosphorylation in Human M2 Macrophages

**Figure 6:**
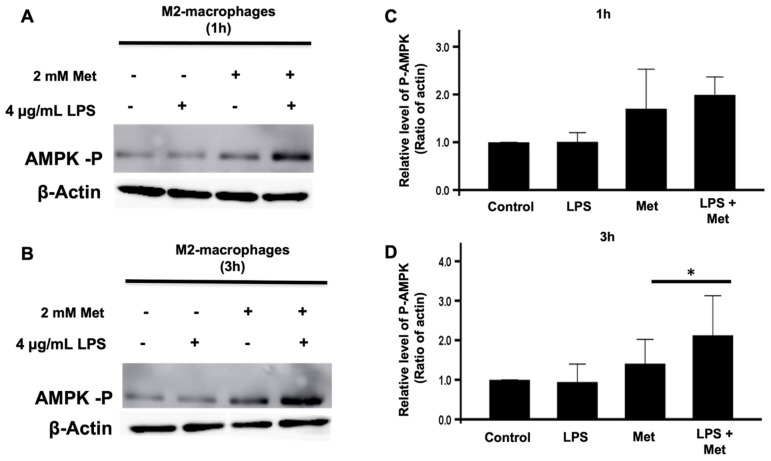
Effect of metformin on the phosphorylation of AMPK in M2 macrophages. M2 macrophages were incubated in the absence or presence of metformin (2 mM), then stimulated with LPS (4 µg/mL) for 1 (**A**) and 3 h (**B**). The phosphorylation of AMPK and the presence of actin were evaluated by SDS-PAGE and Western blot using specific antibodies. The corresponding bands for 1 hour (**C**) and 3 hours (**D**) from three experiments were quantified and expressed as mean ± SD (n = 3, * *p* < 0.05).

Metformin is known to activate AMPK in different cells [5,10,11,12]. Therefore, we next evaluated the effect of metformin on the phosphorylation of AMPK on its Thr172 residue by SDS-PAGE and Western blotting. This residue is located on its α catalytic subunit and reflects AMPK’s activation status. We considered M2 macrophages incubated without metformin and LPS as the control group. Our results clearly show that the treatment of these cells with metformin during 1 and 3 h induced the phosphorylation of AMPK, while LPS alone had no effect (Figure 6A,B). Further, the phosphorylation of AMPK was dramatically enhanced in the presence of LPS and metformin. These results suggest that metformin induced AMPK activation to inhibit several functions, including ROS production in M2 macrophages.

### 3.6. AMPK Plays the Role of a Brake of ROS Production by Human and Mice Phagocytes

**Figure 7:**
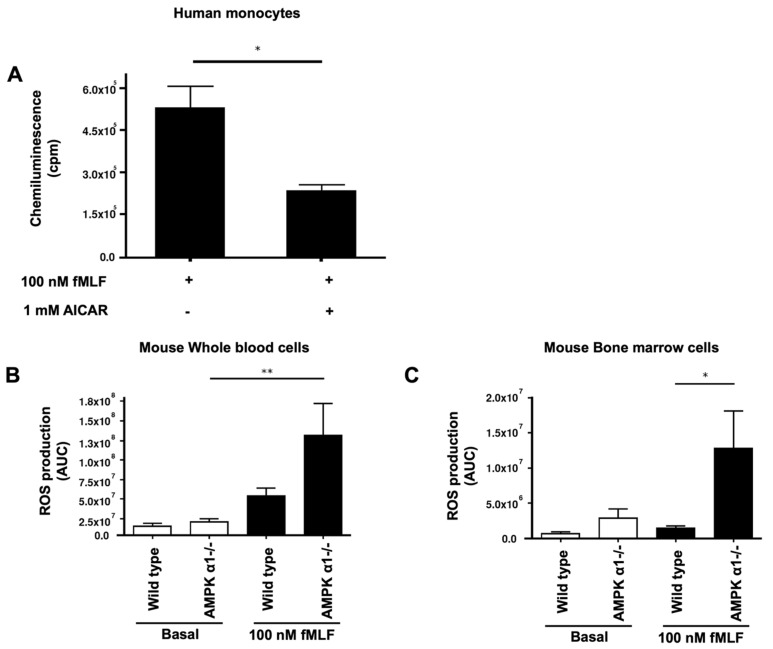
Effect of AMPK activation in human isolated monocytes and AMPK depletion in murine whole blood and bone marrow cells on ROS production. Human monocytes were incubated in the absence or the presence of AICAR (1 mM), stimulated by fMLF (100 nM), and ROS production was measured by luminol-amplified chemiluminescence (**A**). ROS production of whole blood cells (**B**) and bone marrow cells (**C**) isolated from of wild-type mice and AMPKα1-/- mice increased in the presence of fMLF (100 nM). A stronger response to fMLF was observed with AMPKα1-/- versus wild-type cells. The induced luminol-amplified chemiluminescence was measured using the luminometer for 15 min. Data are mean of chemiluminescence (n = 3). * *p* value < 0.05 and ** *p* value < 0.01.

To investigate if, indeed, AMPK could inhibit ROS production in phagocytes, we first used 5-aminoimidazole-4-carboxamide ribonucleotide (AICAR), a known more potent AMPK activator than metformin [48,49]. We incubated human monocytes with 1 mM AICAR for 45 min and stimulated them with *N*-formyl-methionyl-leucyl-phenylalanine (fMLF), a potent inducer of ROS production. ROS production was measured by luminol-amplified chemiluminescence and results show that AICAR significantly decreased ROS production in fMLF-stimulated monocytes (Figure 7A). Second, and to further establish the association between AMPK and ROS production, we used whole blood cells and bone marrow cells collected from wild-type (WT) and AMPKα1-/- mice. Results show that blood cells and bone marrow cells from wild-type mice produced ROS in response to fMLF stimulation (Figure 7B,C). However, and most importantly, results show that blood cells and bone marrow cells from AMPKα1-/- mice produced a higher level of ROS in response to fMLF (Figure 7B,C). These results suggest that the activation of AMPK can block ROS production in cells and could explain the inhibitory effect of metformin on ROS production.

## 4. Discussion

In the present study, we investigated the potential anti-inflammatory effect of metformin by studying the polarization of human monocytes into M1/M2 macrophages and on essential inflammatory mechanisms, such as ROS production by macrophages. We found that metformin is a potent modulator of human monocyte polarization into macrophages by inhibiting the proinflammatory M1 phenotype. We also demonstrated that metformin decreased ROS production in an AMPK-dependent manner in stimulated M2 macrophages.

In order to explore the effect of metformin on the polarization of monocytes into macrophages, we elaborated an in vitro model to differentiate freshly isolated human monocytes into M1 and M2 macrophages. We demonstrate that metformin significantly decreases the polarization of monocytes into M1 macrophages while maintaining their polarization into M2 macrophages. The overall increase in M2 versus M1 macrophages is in agreement with others who mostly studied rodent models and showed that metformin decreased M1 macrophages, with a concomitant increase in M2 macrophage polarization [50,51,52,53]. In a cancer model, it was suggested that metformin increased M1 and decreases M2-like macrophages [54]. The mechanism is unclear and may involve some conditions present in the tumor microenvironment. However, metformin, like AICAR, is an established AMPK activator that is involved in selectively driving M2 macrophage polarization in an inflammatory environment in vitro and in vivo. Indeed, Sag et al. reported that the constitutive activation of AMPKα1 form in the B6J2 macrophage mouse cell line induced the promotion of anti-inflammatory-like macrophage polarization [55]. Interestingly, mouse and human macrophages express predominantly AMPKα1 versus AMPKα2, suggesting that metformin could promote human M2 macrophage polarization. Using a monocytic THP1 cell line, Vasamsetti et al. showed that metformin inhibited monocyte to M1 differentiation via AMPK [56]. The novelty of our study is the use of freshly isolated human monocytes from healthy donors and the comparison of the effect of metformin on monocyte differentiation into M1 and M2 macrophages. It is clear that metformin inhibits M1 macrophage generation, while maintaining or favoring M2 macrophage generation, thus conferring the anti-inflammatory effect. Analysis of the populations of monocytes and macrophage types in patients treated with metformin will be performed to further verify these data in vivo.

Macrophages at the sites of infection can produce ROS and contribute to an oxidative stress. We next wanted to identify whether metformin could regulate M2 macrophage ROS production. Therefore, M2 macrophages’ ROS production was induced by the established agonist LPS in the absence and presence of metformin. Our results showed that metformin significantly inhibited LPS-induced ROS production in M2 macrophages, further extending another protective role of metformin. However, a possible cytotoxic effect of metformin on monocytes and macrophages could account for its action. In this context, we did not observe any cytotoxic effect of metformin, thus confirming that the significant decrease in M1 macrophage polarization and the decrease in ROS production in LPS-induced M2 macrophages were not due to cell death, but rather to a metformin-dependent mechanism. In order to gain insight on the inhibitory effect of metformin on ROS production by M2 macrophages, we investigated if metformin could alter the expression of NOX2 subunits. We found that metformin, like LPS, did not have such an effect on the NOX2 subunits (gp91^phox^, p22^phox^, p67^phox^, p47^phox^). Further, it is well established that the activation of the NOX complex depends on the phosphorylation of the regulatory cytosolic subunit p47^phox^. In fact, MAP kinases (ERK 1/2 and p38) are converging pathways, which phosphorylate p47^phox^ on its 345-serine residue located within a consensus sequence recognized by these kinases [38,39,40]. This will then induce the movement and assembly of all cytosolic subunits, with the catalytic subunits at the membrane forming the active NOX complex [30]. Therefore, we assessed the effect of metformin on the phosphorylation levels of the p47^phox^ regulatory subunit and the MAP kinases, ERK1/2 and p38, in M2 macrophages. Metformin did not affect the phosphorylation levels of p47phox on Ser345 or ERK 1/2 and p38 MAP kinases.

The inhibitory mechanism of metformin on M2 macrophage ROS production was further explored by studying the phosphorylation of AMPK. Metformin is known to activate AMPK, allowing it to regulate the energetic cell status [16] and possibly the production of ROS by phagocytes [57]. Thus, we evaluated if metformin could affect AMPK phosphorylation levels in M2 macrophages and found that it clearly induced AMPK phosphorylation. Interestingly, LPS was ineffective in inducing the phosphorylation of AMPK, in agreement with studies performed with human bone-marrow-derived macrophages performed with 1 h and 3 h incubation [55]. Moreover, we show that the effect of metformin on AMPK phosphorylation was potentiated when incubated in the presence of LPS. Thus, it is possible that phosphorylated AMPK induced by metformin could participate in our observed inhibition of ROS production in the presence of LPS. To further demonstrate the inhibitory role of AMPK on ROS production, we used AICAR, another positive effector of AMPK phosphorylation. In fact, in cell-based assays, AICAR is a known standard positive control used for the activation of AMPK [48,49]. Once internalized by the cell, adenine kinase phosphorylates AICAR and turns it into 5-aminoimidazole-4-carboxamide ribonucleotide (also known as *Z*-nucleotide monophosphate (ZMP)), which is an AMP analogue. Both the phosphorylation of AMPK and its binding with a nucleotide lead to its activation [58]. Therefore, we incubated AICAR with monocytes and measured ROS production. We show that AICAR significantly decreased ROS production in fMLF-induced human isolated monocytes, confirming that AMPK activators (metformin and AICAR) can decrease ROS production in activated immune cells. It is worth noting that LPS and fMLF alone are known inducers of cellular ROS production in macrophages [59]. To bring additional proof on the inter-relationship between AMPK and ROS production, we used AMPKα1-/- knockout mice. Our findings show that the depletion of AMPKα1 significantly increased ROS production in fMLF-stimulated murine whole blood and bone marrow cells. To summarize, the activation of AMPK decreases ROS production, while the depletion of AMPK counteracts this effect in induced immune cells. In addition, metformin’s treatment increases AMPK phosphorylation level in induced immune cells. Taken together, we suggest that metformin may exhibit its antioxidant and anti-inflammatory effects by lowering ROS production in stimulated immune cells in an AMPK-dependent manner.

**Figure 8:**
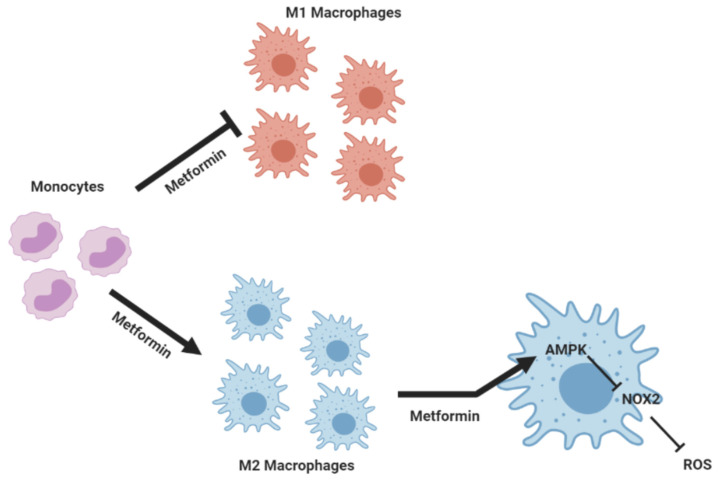
Schematic representation of metformin’s action on macrophage polarization, showing its anti-inflammatory and antioxidant effects on macrophages. Metformin inhibits the polarization of M1 macrophages while favoring M2 macrophages. In addition to this effect, metformin dampens ROS production by these cells. Metformin may exert its anti-inflammatory and antioxidant effects through the reduction in NOX2-induced ROS production in an AMPK-dependent manner.

In addition to its antidiabetic effect, metformin exhibits anti-inflammatory, antioxidant, and anticancer effects [2,3,4]. Metformin is a known pharmacological activator of AMPK. AICAR, another pharmacological activator of AMPK, enhances the efficacy of rapamycin, the mTORC1 inhibitor, to kill human cancer cells by regulating phospholipase D (PLD) activity [21]. The effect of metformin on the mTOR/PLD axis in monocytes and macrophages will be investigated. These data suggest that pharmacological activation of AMPK by metformin could be an interesting strategy in cancer treatment and other diseases treatment. Our findings are schematically summarized in Figure 8. In a general context of inflammation, metformin might help in ceasing the inflammatory status by inhibiting the polarization of human monocytes into M1 macrophages while favoring their polarization into M2 macrophages. In addition, our work suggests that metformin exerts its anti-inflammatory and antioxidant effects through decreasing ROS production in an AMPK-dependent manner.

## Data Availability

Not applicable.

## Abbreviations

AICAR	5-Aminoimidazole-4-carboxamide ribonucleotide
AMPK	adenosine monophosphate activated protein kinase
CD	cluster of differentiation
fMLF	*N*-formyl-methionyl-leucyl-phenylalanine
GM-CSF	granulocyte macrophages colony stimulating factor
LPS	lipopolysaccharide
M-CSF	colony-stimulating factor
NBT	nitro blue tetrazolium
NOX	nicotinamide adenine dinucleotide phosphate oxidase
PBMC	peripheral blood mononuclear cells
ROS	reactive oxygen species
